# Mr. Cuming, on Vaccine Prophylaxis

**Published:** 1804-09-01

**Authors:** Ralph Cuming

**Affiliations:** Romsey, Hants


### *To the Editors of the Medical and Physical Journal.*

GENTLEMEN,

TO the man of letters, philosopher, and philanthropist, a mine of pleasure must display itself, when he casts his eye over the wide field of medical discussion and research, which has been entered of late, with so much candour; where we find those branches of the profession, who have not been honoured with the distinguishing mark of M. D. daring to call in question, opinions advanced by the very fathers of medicine, the first rate physicians of their day. Your Journal is the medium through which much useful information has been given by the exertions of liberal and disinterested men; men who are not wrapped up in unchristian-like apathy, and who do not conceal their talents in a napkin, or throw cold water on a subject, meriting the warmest support of investigation from every man, whose heart throbs with benevolent desires.

If my endeavours may tend to ward off a weapon, which has been lately wielded, against a discovery inferior to none, in point of the blessings and advantages which must ultimately accrue from it, I shall be glad if you will find a place for the insertion of this paper.

There is a man in this neighbourhood, who has gained a livelihood by following the occupation of itinerant inoculator, and is distinguished with the epithet of Inoculating Doctor, who has variolated his thousands, *and tens of thousands!* In the course of his practice amongst the peasantry, he has frequently been disappointed in infecting many of his applicants, and was perfectly unable to account for his failures, until many of them talked of having formerly had the cow-pox. At length, the theme of the cow-pox so often rang in his ears, as to induce him to suppose, that there really was some truth in what he had heard so often asserted; namely, that those who had been infected with it, could never take the small-pox. After this information, when he met with persons he could not infect, he always asked if they had had the cow-pox; the answer being in the affirmative, he has declared his inability to give them the small-pox, and their security from it to have arisen from the cause already assigned. Yet some people, assenting to this fact, observe, when the matter is taken from the human subject, there is reason to suppose its properties are so changed, that it soon loses its original

 effects,

‹ ffects, and may render the constitution again susceptible of the variolous infection, after preserving it for a few years, which some have said, since the publication of Mr. Goldson's pamphlet, appears to be the case. Such ideas from individuals who never made the laws of animal economy their study, are very excusable; but I cannot restrain my astonishment, when I find Mr. G. the first to broach and support this noxious doctrine, which has already rivetted many to their unfounded prejudices, and hermetically sealed every avenue to reason and conviction; such are the baneful effects of this pernicious pamphlet. Does not every one, whose knowledge is not extremely circumscribed, know, that specific poisons or contagions are invested with the peculiar power of assimilating or converting the humours of the body to their own particular properties; this is an incontrovertible fact, which daily experience teaches us, and which has been known for centuries; but the manner in which this change is effected, is one of the many hidden mysteries of Nature, which man, in his finite capacity, may never be able to comprehend; yet some are so sceptical as to doubt every thing they cannot understand; with such, it is hard to decide, whether pride or folly is most predominant. If the above proposition is doubted, I would ask, how does the rabies canina infect every animal that we know of, without losing one atom of its virulence? The lues venerea also proves the truth of the position in the human body. It has been fully proved, that the infection of the cow-pox has been reciprocally given and taken between the cow and the milker, that one milker has given it to another; yet, notwithstanding every possible variety and change, from subject to subject, its properties remain the same. Mr. G. observes, he wishes the business to be calmly and quietly discussed, without exciting controversy; or something to this effect. Can he suppose that the circulation of a pamphlet, freighted with such poisonous materials, and wafted through the world by the popular gale of prejudice and ignorance, should escape censure? or that people are to remain silent, and behold with an eye of apathy that which hath done irreparable injury to the cause of humanity? Let me ask, what were the motives which induced Mr. G. to publish his book? Had they proceeded from a truly benevolent source, why did he not wait patiently until the necessary enquiries were instituted, and answers had been received respecting what he terms failures of vaccination? then purity of motive could never have been doubted: it is easy to make make professions, but it is from actions that correct opinions are to be deduced.

The late Dr. Brown, a naval surgeon of considerable repute, who had been practising some time at Portsmouth, informed me that he was called to witness *a Portsmouth failure* of the cow-pox, wherein subsequent inoculation with variolous matter had produced the disease. You may guess what was his surprize, when he, in company with others of the profession, saw the child; he enquired where the eruption was? (the child's arm being denuded) but lo! it had fled; he was answered it was very visible yesterday. How the tide of their unnatural joy must soon have ebbed! But I beg you to observe, that this is *une maladie nouvelle*, which may aptly be termed *the Portsmouth variola ephemera*. Dr. Brown's reasoning on the subject did credit to him, and the truth of his opinion has been confirmed and experienced by Mr. Ring. He said it was easy to conceive, that virulent matter introduced into the system, might produce local inflammation, and even constitutional derangement, yet it was evident that the asseverations of the gentlemen were not substantiated; for nothing like the small-pox was the result of the subsequent inoculation. Mr. G. states, that he infected patients with the variolous matter taken from those who had been previously vaccinated; I mention this circumstance on the supposition that the vaccine matter previously employed had been genuine, which it certainly was not: I say, let him avail himself of this, his favorite and *mighty* argument, and observe how weak and futile it will appear. Does not every one know that nurses and mothers, who suckle their children that are ill of the small-pox, frequently have an eruption of pox on their breasts, when they themselves have formerly had the complaint? and can it be doubted, whether matter from this source will produce the idiopathic disease? Then, from parity of reasoning, matter introduced into the arm after vaccination, may be employed for a similar purpose, and will exert its accustomed influence over the body, which is not prepared to resist it, by means of the vaccine antidote, that will

> For ages, on the wings of love, be born,
> To bless the rich, the wretched, and forlorn;
> Whilst millions shall to JENNER raise,
> Throughout the world, a monument of praise.

I have many proofs in my own possession of the prophylactic powers of the vaccine virus; but it is not necessary to make further mention of proofs, which for years

P 2 have have been teeming on the public in accumulated numbers, or to take up any more of your Journal, which will be better employed in prosecuting medical research, in matters that have not been proved with that precision and certainty which the subject of this paper will appear to have been, to any, save the sceptic, whose perverted reason only views objects for the sake of inverting them.

I am, &c.

*Romsey, Hants, Aug.* 6, 1804. RALPH CUMING.

